# Cooking smoke and respiratory symptoms of restaurant workers in Thailand

**DOI:** 10.1186/s12890-017-0385-7

**Published:** 2017-02-17

**Authors:** Chudchawal Juntarawijit, Yuwayong Juntarawijit

**Affiliations:** 1 Department of Natural Resource and Environment, Faculty of Agriculture, Natural Resource and Environment, Naresuan University, 99 Moo 9, Thaphao sub-district, Amphur Muang, Phitsanulok, 65000 Thailand; 2 Faculty of Nursing, Naresuan University, Phitsanulok, Thailand

**Keywords:** Cooking fumes, Kitchen smoke, Restaurant workers, Restaurant health and safety, Respiratory health, Health symptoms

## Abstract

**Background:**

Restaurant workers are at risk from exposure to toxic compounds from burning of fuel and fumes from cooking. However, the literature is almost silent on the issue. What discussion that can be found in the literature focuses on the potential effects from biomass smoke exposure in the home kitchen, and does not address the problem as occurring in the workplace, particularly in restaurants.

**Methods:**

This was a cross-sectional survey of 224 worker from 142 food restaurants in the Tha Pho sub-district of Phitsanulok, a province in Thailand. The standard questionnaire from the British Medical Research Council was used to collect data on chronic respiratory symptoms, including cough, phlegm, dyspnea, severe dyspnea, stuffy nose in the participating workers. Data on their health symptoms experienced in the past 30 days was also asked. A constructed questionnaire was used to collect exposure data, including type of job, time in the kitchen, the frequency of frying food, tears while cooking (TWC), the type of restaurant, fuel used for cooking, the size and location of the kitchen, and the exhaust system and ventilation. The prevalence of the symptoms was compared with those obtained from 395 controls, who were neighbors of the participants who do not work in a restaurant.

**Results:**

In comparison to the control group, the restaurant workers had twice or more the prevalence on most of the chronic health symptoms. Men had a higher risk for “dyspnea”, “stuffy nose” and “wheeze” while women had higher risk of “cough”. A Rate Ratio (RR) of susceptibility was established, which ranged from 1.4 up to 9.9. The minimum RR was for women with “severe dyspnea” (RR of 1.4, 95%CI 0.8, 2.5) while the men showed the maximum RR of 9.9 (95%CI 4.5–22.0) for “wheeze”. Possible risk factors identified were job description, job period, size of restaurant, kitchen location, type of cooking oil, hours of stay in the kitchen area, number of fry dishes prepared, frequency of occurrence of TWC, and additional cooking at home. Working for 6–10 year increased the risk of “cough” with an Odd Ratio (OR) of 3.19 (*P* < 0.01) while working for more than 10 years increased the risk of “cough” (OR = 3.27, *P* < 0.01), “phlegm” (OR = 3.87, *P* = 0.01) and “wheeze” (OR = 2.38, *P* = 0.05). Working as a chef had a higher risk of “cough” by 2.33 (*P* = 0.01) as comparing to other jobs. Workers in a relatively large restaurant using 4 or more stoves had increased risk of “wheeze” with OR of 3.81 (*P* < 0.01) and “stuffy nose” with OR of 3.56 (*P* < 0.01). Using vegetable oil increased the risk of “stuffy nose” by 2.94 (*P* < 0.01). Every 10 h of stay in the kitchen area was associated with a minimal increase in the risk of “cough”, “wheeze” and “symptoms in the past 30 days” by 1.15 (*P* = 0.02), 1.16 (*P* = 0.01) and 1.16 (*P* = 0.02), respectively.

**Conclusions:**

Restaurant workers are at risk of respiratory symptoms caused by exposure to toxic compounds from cooking fumes. Job description, job period, size of restaurant, kitchen location, type of cooking oil, hours of stay in the kitchen area, number of fry dishes prepared, frequency of occurrence of TWC, and additional cooking at home were the predictive factors. Workplace Health and Safety protection of restaurant worker is urgently needed and the issue should receive more public attention.

## Background

Running a restaurant is one of the most common businesses with possibly millions of workers involved. However, the health and safety of these workers is often overlooked. Restaurant workers are at risk from exposure to toxic compounds from burning of fuel and fumes from cooking. Nitrogen dioxide, carbon monoxide and particulate matter from fuel combustion may affect the development or exacerbation of asthma, for example [1]. Under high temperatures, degradation of sugar and fat, and pyrolysis of proteins and amino acids, may create respiratory irritant compounds, such as acrolein and formaldehyde [2]. It was been reported that smoke from burning fuel and fumes from cooking contain many toxic substances, some of which are carcinogenic, such as polycyclic aromatic hydrocarbons (PAHs), amines, benzene and formaldehyde [3, 4]. An elevation of aerosols is also commonly reported during cooking [5], depending on the type of oil, temperature and type of food [6]. Among the different types of cooking, deep frying emits the most PM2.5, followed by frying, stir frying, boiling and steaming [7]. A survey of restaurants in several locations found harmful levels of various pollutants. Measurement of air samples in a Korean style restaurant in Hong Kong, for instance, found PM10, PM2.5 and carbon monoxide (1 h average) at the level of 1.44, 1.17 and 15.10 mg/m3 [8]. Benzene, toluene, methylene chloride and chloroform were also detected. Siao et al. [9] reported elevated levels of PM2.5 and PAH in Chinese and Indian style restaurants. Researcher have also reported elevated levels of genotoxicant (8-OHdG) in male kitchen workers [10]. Women (and presumably men also) who cook at home are exposed to toxic compounds [11].

Most available literature has reported a positive association between cooking fumes and lung cancer, with some predictive factors, including job description, type of cooking oil, exhaust hood, oil temperature, deep fry and stir fry cooking [12, 13]. The risk of lung cancer per 10 dish-year among those who deep fry, fry and stir fry was found to be 2.56, 1.47 and 1.12, respectively [14]. Use of biomass fuel has also been related to PM2.5 and Cr (VI) and risk of lung cancer [15]. However, there have only been a few studies which focused on the respiratory health of restaurant workers [16]. A study of kitchen workers in Norway reported an increased work related risk of dyspnea, severe dyspnea and other health symptoms among women employees [17]. However, the study also found “working in the kitchen” as the only predictive risk factor. Adewole et al. [18] also reported an elevated risk of cough, chest tightness, congestion and wheezing among kitchen workers in Nigeria. However, a recent study in Hong Kong [19] failed to find a significant difference in the prevalence of respiratory symptoms between restaurant worker cooking with gas and those cooking with electricity. Therefore, it is suggested that more studies are needed to identify more risk factors.

This was the purpose of this study, to survey the occurrence of symptoms of work-related health issues among restaurant workers in Thailand, given the unique cooking style found in Thai restaurants, with the intention of identifying a comprehensive set of risk factors that may arise from the different food preparation methods in Thai restaurants, and any other factors relating to the health and even cultural backgrounds of workers in Thai restaurants.

## Methods

Tha Pho is a small rural community with the total area of 50.7 km^2^, and population of 23,130 people (9314 men, 13,816 women). The main occupation of the villagers is rice farming. There are no industrial plants in the district. The only business facility in the community is Naresuan University [20]. This study includes only those food restaurants located outside the university.

By using the consecutive sampling technique, this cross-sectional study was able to recruit 142 food restaurants: 82 serving Thai cuisine à la carte, 23 noodle restaurants, 31 restaurants serving pre-prepared food, and 6 papaya salad restaurants. A brief explanation is warranted here, on the type of restaurants stated, and the implication of each of them in our study. Restaurants serving Thai cuisine cooked many dishes by frying in oil, given the nature of many Thai dishes. In noodle restaurants, the food is prepared predominantly by boiling in water, with little or no use of oil for cooking. Restaurants serving pre-prepared food, which then laid out the various foods in bain marie’s or other warming devices, usually did not prepare the food on-site, so no cooking took place in that restaurant. Finally, restaurants serving papaya salad as their usual product usually cooked very little of their ingredients by oil or boiling water. The relative prevalence of oil cooking did affect our results, as will be discussed further.

All workers in each restaurant were interviewed by 9 village health volunteers. Prior to collection of the data, the volunteers were informed of the study purposes and the data collection procedure, which used a questionnaire, which was also carefully discussed. The survey was conducted in the period of October to December 2014, winter time in Thailand, during which season average daily temperatures are about 30 °C with minimal rainfall. Weather conditions therefore did not affect the health status of the workers. Data regarding respiratory health, type of job and kitchen smoke exposure were collected. Duration in the kitchen was measured with “hours per week working in a kitchen” (the number of hours per working day multiplied by the number of days worked per week in a kitchen). Frying frequency was measured by “fry-dishes prepared per week” (the number of fry-dishes per day multiplied by number of day frying food in a week). Information on tears while cooking (TWC), the type of restaurant, fuel used for cooking, the size and location of the kitchen, the exhaust system and ventilation was also collected.

The questions on respiratory health were based on a standard questionnaire for chronic lung disease developed by the British Medical Research [21] and American Thoracic Association (ATS-DLD-78 questionnaire) [22]. Respiratory health data elicited included prevalence of cough, phlegm, dyspnea, severe dyspnea, wheeze and other related symptoms of respiratory problems. Participants were attributed with a “cough” if they reported chronic cough with or without phlegm for as much as 4 to 6 times a day, 4 or more days out of the week. “Phlegm” was reported as having only sputum but no cough for as much as twice a day, 4 or more days out of the week. “Wheeze” referred to those who breathe with whistling sound. “Dyspnea” referred to those with any of shortness of breath. “Severe dyspnea” is those with shortness of breath even when undertaking ordinary activities. “Stuffy nose” referred to other who with stuffy nose with a cold. Data on the prevalence of respiratory health symptoms occurring during the past 30 days (“symptoms in 30 days”) was also collected. The criteria symptoms included in this survey were coughing, having sputum, sore throat, shortness of breath or chest tightness, wheezing, nasal irritation or nasal congestion, allergic symptom and having a cold.

The control group consisted of neighbors who did not work in a restaurant and who lived at least 50 m away from a restaurant. For each study subject, 2 unmatched control subjects were interviewed using the same questionnaire. In this study, data from 224 cases and 395 controls were included for statistical analysis.

Data was analyzed using SPSS v.22 for Windows and OpenEpi v.3.01 (online). The prevalence rate was computed using descriptive statistics. Prevalence and RR of health symptoms in each gender among the study subjects and the control group were compared using Cochran’s and Mantel-Haenszel statistics, controlling for age and tobacco consumption. Predictive risk factors were identified using binary logistic regressions adjusted for age and tobacco consumption.

## Results

The study population was primarily women (67% in study group and 56% in control group) with a similar mean age of around 40 years in both the study and control groups (Table 1). Most of them were chefs (65.6%) and the rest working as both assistant chef and waitress. In the study group, the average time working in this occupation and the average time staying in this particular type of work (i.e. cook, waiter) was similar, 7 years. The average time staying in a kitchen was 41 h/week for women participants and 32 h/week for men, which was much higher than for the control group (8 h/week for women and 4 h/week for men). Compared to men, women cooked fried food more often with an average number of fried dishes per week of 3448.2 for the women, and 1801.6 for the men. Smoking was more common among men, with 44.0% in the study group and 53.5% in the control group, while only 3.4% of the women in the study group smoked, compared to 3.2% of the women in the control group. Between 64.5% and 91.0% of all participants reported that they also cook at home.

**Table 1:** Data on demography, work environment and cooking activity

	Restaurant workers		Control group	
	Women (*n* = 149)	Men (*n* = 75)	Women (*n* = 219)	Men (*n* = 172)
Age	40.9 ± 11.3^a^	41.9 ± 11.9	44.4 ± 15.8	45.2 ± 17.5
< 40 year	65 (43.6)	29 (38.7)	81 (36.8)	62 (36.0)
≥ 40 year	84 (56.4)	46 (61.3)	138 (62.7)	110 (64.0)
Job description				
Chef	147 (65.6)		-	
Other^b^	77 (34.4)		-	
Years in this occupation	7.3 ± 5.1	7.6 ± 6.9	-	-
0–5 year	107 (47.8)		-	
6–10 year	75 (33.5)		-	
> 10 year	42 (18.8)		-	
Years in this type of job	6.7 ± 4.9	5.9 ± 6.5	-	-
Hour per week in a kitchen	41.4 ± 25.7	32.2 ± 25.7	8.0 ± 6.7	4.1 ± 5.3
Fry dishes per week^c^	3448.2 ± 24,264.5	1801.6 ± 11,902.0	14.2 ± 19.5	7.8 ± 13.2
Smoker^c^	5 (3.4%)	33 (44.0%)	7 (3.2%)	92 (53.5%)
People cooking at home	136 (91.3%)	65 (86.7%)	187 (85.0%)	111 (64.5%)
Work environment and cooking activity among case group				
Number of subject in each type of restaurant (*n* = 224)				
Thai cuisine à la carte	129 (57.6)		-	
Noodle restaurant	35 (15.6)		-	
Pre-prepared food	49 (21.9)		-	
Papaya salad restaurant	11 (4.9)		-	
Number of stove in each restaurant				
1	18 (8.0)		-	
2	92 (41.1)		-	
3	47 (21.0)		-	
4	34 (15.2)		-	
5	18 (8.0)		-	
6	4 (1.8)		-	
7	8 (3.6)		-	
8	2 (0.9)		-	
12	1 (0.4)		-	
Having separated kitchen room				
Yes	179 (79.9)		-	
No	45 (20.1)		-	
Having ventilation hood				
Yes	110 (49.1)		-	
No	114 (50.9)		-	
Type of cooking fuel				
LPG	160 (71.4)		-	
Biomass	14 (6.3)		-	
Electricity	2 (0.9)		-	
LPG and biomass	46 (20.5)		-	
LPG and electricity	2 (0.9)			
Type of cooking oil				
Soybean oil	13 (5.8)		-	
Palm oil	165 (73.7)		-	
Lard	46 (20.5)		-	
Number of fry dish per week (*n* = 201; mi*n* = 0, max = 2400; mean ± SD = 441 ± 456, media*n* = 350)				
0–1	50 (22.3)		-	
2–350	56 (25.0)		-	
351–745	45 (20.1)		-	
≥ 746	50 (22.3)		-	
Cooking at home				
Every day	135 (60.3)		-	
Some days	66 (29.5)		-	
Not do	23 (10.3)		-	

^a^ Quantitative data are presented as mean ± SD; Category data are presented in number (percent)

^b^ Other referred to those who working as a both chef assistant and waitress

^c^ Significantly different between women and men

Most of the participants worked in restaurants serving Thai cuisine à la carte (57.6%) (Table 1). Most of the restaurants are small in size, 70% having 1–3 cooking stoves, 71.4% using LPG for cooking, and 79.5% using vegetable oil for cooking. Up to 20% of the restaurants operated without a separated kitchen room, and 50% without a ventilation hood. Most of the study subjects reported frying food with the median number of 350 fry-dish per week. Both study and control group reported cooking at home, with 60.3% cooking every day and 29.5% cooking some days.

### Prevalence of respiratory symptoms

The restaurant workers surveyed had a significantly higher prevalence of all chronic respiratory symptoms than the control group, except for severe dyspnea (Table 2). The three symptoms with the highest prevalence among women were dyspnea, stuffy nose and cough; and among men were stuffy nose, wheeze and cough. The RR of the participants and the control group, after adjusting for age and smoking, was between 1.4 (95%CI 0.8, 2.5) for severe dyspnea in women and 9.9 (95%CI 4.5–22.0) for wheeze in men. Women were at greater risk of “cough”, “phlegm” and “wheeze”, with men at greater risk of “wheeze”, “stuffy nose” and “dyspnea”.

**Table 2:** Prevalence (per 100 population) of chronic respiratory symptoms among the restaurant workers and the control group

Health symptoms	Women			Men		
	Restaurant workers	Controls	RR^a^ (95% CI)	Restaurant workers	Controls	RR (95% CI)
Chronic symptoms						
Dyspnea	52.3	27.5	1.9 (1.5–2.5) ^b^	30.7	10.5	3.1 (1.7–5.7) ^b^
Stuffy nose	45.8	27.8	1.7 (1.3–2.2) ^b^	48.0	16.9	3.1 (2.0–4.8) ^b^
Cough	32.5	9.1	3.7 (2.3–5.9) ^b^	32.0	16.3	2.0 (1.3–3.1) ^b^
Wheeze	25.5	11.5	2.1 (1.4–3.4) ^b^	38.7	3.5	9.9 (4.5–22.0) ^b^
Phlegm	14.4	5.8	2.5 (1.3–4.8) ^b^	25.3	8.7	2.7 (1.5–5.0) ^b^
Severe dyspnea	11.4	8.3	1.4 (0.8–2.5)	6.7	6.4	1.4 (0.9–3.4)
Symptoms in the past 30 days						
Coughing	42.5	38.1	1.1 (0.9–1.5)	54.7	43.0	1.3 (1.0–1.8) ^b^
Having a cold	25.2	22.2	1.1 (0.8–1.6)	29.3	12.8	2.4 (1.4–4.2) ^b^
Sore throat	25.2	20.8	1.2 (0.9–1.8)	34.7	16.9	2.1 (1.3–3.4) ^b^
Having sputum	23.8	19.9	1.2 (0.8–1.8)	33.3	16.9	2.2 (1.3–3.5) ^b^
Allergic symptom	13.1	11.7	1.1 (0.6–1.9)	10.4	6.9	1.4 (0.6–3.5)
Shortness of breath	10.3	4.5	2.1 (1.0–4.4) ^b^	11.4	5.1	2.2 (0.8–5.9)
Wheezing	9.2	6.3	1.5 (0.7–3.0)	15.6	3.4	4.8 (1.8–12.7) ^b^
Nasal irritation	5.9	4.5	1.3 (0.5–3.1)	7.4	4.5	1.5 (0.6–3.8)
Having any one symptoms	63.1	49.5	1.3 (1.1–1.6) ^b^	73.3	50.0	1.6 (1.3–1.9) ^b^

^a^ Pooled risk ratio (RR) analyzed using Cochran’s Mantel-Haenszel statistic adjusted for age (<40 vs ≥40) and smoking (ever smoked vs. never smoke) and correct zero count by adding 1 to each of the 4 cells

^b^ Significantly different between women and men

It was also found that the restaurant workers displayed a greater number of symptoms in the past 30 days than the control group. The symptoms with significant RR were shortness of breath, coughing, having sputum, sore throat, wheezing and having a cold (Table 1). Of the female participants, 42.5% had a cough, 25.2% had a cold and 25.2% had a sore throat. The men’s most common symptoms were coughing (54.7%), sore throat (34.7%) and having sputum (33.3%).

### Risk factors for health symptoms

For factors creating chronic risks to the health of restaurant workers are presented in Table 3. When compared with the other types of restaurants, working in an à la carte restaurant showed an increased risk of “cough” and “phlegm” but the related OR were not statistically significant. Comparisons between the various different types of restaurant did not show any significant association between types of restaurant and prevalence of chronic symptoms. The role of chef showed a higher risk of “cough” and “dyspnea”. Working in a restaurant with a separated kitchen room was associated with “wheeze” and “stuffy nose”, while workers in a large restaurant with more than 3 stoves had a risk of “wheeze” 3.82 times those in small shops, with “stuffy nose” being 3.56 times those in small shops. Using vegetable oils (soybean and palm oil) showed a higher risk of “stuffy nose” than when lard (pig fat) was used. TWC every day can predict the risk of “cough”, “wheeze” and “stuffy nose” while TWC on some days can predict all symptoms except “wheeze” and “severe dyspnea”. TWC can also predict the trend of risk for “cough” and “stuffy nose”.

**Table 3:** Odds Ratios (OR) with p values for predictors for chronic respiratory symptoms of restaurant workers

Predictors	Cough	Phlegm	Wheeze	Dyspnea	Severe dyspnea	Stuffy nose
À la carte restaurant vs. other type	1.63 (0.91–2.93) *P* = 0.10	1.99 (0.92–4.29) *P* = 0.08	0.90 (0.50–1.64) *P* = 0.74	0.69 (0.40–1.18) *P* = 0.18	0.59 (0.24–1.44) *P* = 0.25	0.78 (0.46–1.32) *P* = 0.35
Noodle vs à la carte restaurant	1.55 (0.39–6.17) *P* = 0.54	2.93 (0.34–25.39) *P* = 0.33	1.04 (0.25–4.33) *P* = 0.96	0.55 (0.16–1.91) *P* = 0.35	NA	0.66 (0.19–2.27) *P* = 0.51
Pre-prepared food vs à la carte restaurant	0.57 (0.12–2.82) *P* = 0.49	1.08 (0.10–12.33) *P* = 0.95	0.94 (0.19–4.56) *P* = 0.94	0.82 (0.21–3.21) *P* = 0.77	NA	0.45 (0.11–1.80) *P* = 0.26
Papaya salad vs à la carte restaurant	1.27 (0.29–5.49) *P* = 0.75	1.86 (0.20–17.75) *P* = 0.59	1.35 (0.30–6.04) *P* = 0.70	0.74 (0.20–2.80) *P* = 0.66	NA	1.27 (0.34–4.77) *P* = 0.73
Working 6–10 year vs 0–5 years	3.19 (1.60–6.35) *P* < 0.01^*^	1.77 (0.72–4.34) *P* = 0.21	1.59 (0.79–3.21) *P* = 0.20	1.43 (0.77–2.66) *P* = 0.25	1.07 (0.34–3.30) *P* = 0.91	0.95 (0.52–1.74) *P* = 0.86
Working >10 year vs 0–5 years	3.27 (1.37–7.83) *P* < 0.01^*^	3.87 (1.35–11.07) *P* = 0.01^*^	2.38 (1.01–5.60) *P* = 0.05^*^	2.64 (1.19–5.89) *P* = 0.02^*^	2.83 (0.87–9.20) *P* = 0.08	0.89 (0.40–1.95) *P* = 0.76
Chef vs others	2.33 (1.21–4.49) *P* = 0.01^*^	1.30 (0.60–2.82) *P* = 0.51	1.89 (0.97–3.71) *P* = 0.06	2.12 (1.18–3.81) *P* = 0.01^*^	1.41 (0.52–3.81) *P* = 0.50	1.51 (0.85–2.68) *P* = 0.16
Having vs not having separated kitchen room	1.02 (0.50–2.07) *P* = 0.96	1.03 (0.41–2.59) *P* = 0.95	5.40 (1.82–16.06) *P* = <0.01^*^	1.05 (0.54–2.03) *P* = 0.89	1.65 (0.46–5.85) *P* = 0.44	2.48 (1.22–5.05) *P* = 0.01^*^
Large vs. small restaurant^b^	1.28 (0.70–2.34) *P* = 0.43	0.91 (0.41–2.01) *P* = 0.82	3.82 (2.01–7.25) *P* < 0.01^*^	0.88 (0.50–1.58) *P* = 0.68	1.36 (0.54–3.42) *P* = 0.52	3.56 (1.94–6.53) *P* < 0.0 1^*^
Vegetable oil vs. lard^a^	1.68 (0.79–3.57) *P* = 0.18	1.02 (0.42–2.48) *P* = 0.97	1.93 (0.86–4.32) *P* = 0.11	1.43 (0.73–2.78) *P* = 0.30	0.92 (0.32–2.66) *P* = 0.87	2.94 (1.43–6.07) *P* < 0.01^*^
Having vs. not having ventilation hood	1.55 (0.88–2.74) *P* = 0.13	1.97 (0.94–4.12) *P* = 0.07	1.33 (0.73–2.40) *P* = 0.35	1.50 (0.88–2.56) *P* = 0.14	0.83 (0.34–2.03) *P* = 0.69	1.10 (0.65–1.86) *P* = 0.73
Per 10 h increase in working in kitchen each week	1.15 (1.02–1.29) *P* = 0.02^*^	1.09 (0.95–1.24) *P* = 0.22	1.16 (1.03–1.32) *P* = 0.01^*^	1.08 (0.98–1.21) *P* = 0.13	1.12 (0.95–1.32) *P* = 0.16	1.09 (0.98–1.22) *P* = 0.11
Fry 2–350 dishes per week vs. no fry	1.24 (0.48–3.24) *P* = 0.66	2.52 (0.77–8.22) *P* = 0.13	0.50 (0.18–1.31) *P* = 0.16	0.73 (0.33–1.60) *P* = 0.43	1.81 (0.42–7.83) *P* = 0.43	1.26 (0.56–2.84) *P* = 0.57
Fry 351–745 dishes per week vs. no fry	3.03 (1.18–7.79) *P* = 0.02^*^	1.06 (0.26–4.43) *P* = 0.93	1.29 (0.51–3.30) *P* = 0.59	0.60 (0.26–1.38) *P* = 0.23	2.42 (0.56–10.46) *P* = 0.24	2.30 (0.99–5.36) *P* = 0.05^*^
Fry ≥746 dishes per week v. no fry	5.03 (1.96–12.91) *P* < 0.01^*^	4.84 (1.46–15.97) *P* = 0.01^*^	2.67 (1.09–6.58) *P* = 0.03^*^	1.73 (0.76–3.95) *P* = 0.20	1.70 (0.37–7.84) *P* = 0.50	3.23 (1.37–7.60) *P* < 0.01^*^
Cooking every day at home vs. not cook at home	3.98 (1.12–14.23) *P* = 0.03^*^	2.76 (0.58–13.17) *P* = 0.20	6.72 (1.44–31.38) *P* = 0.02^*^	3.64 (1.27–10.40) *P* = 0.02^*^	2.47 (0.31–19.86) *P* = 0.39	5.60 (1.79–17.52) *P* < 0.01^*^
Cooking some days at home vs. not cook at home	2.82 (0.75–10.67) *P* = 0.13	2.16 (0.42–10.98) *P* = 0.36	3.44 (0.70–16.96) *P* = 0.13	2.49 (0.82–7.55) *P* = 0.11	2.54 (0.29–21.97) *P* = 0.40	3.56 (1.08–11.73) *P* = 0.04^*^
TWC every day vs. never TWC	4.60 (1.85–11.44) *P* < 0.01^*^	3.37 (0.83–13.62) *P* = 0.09	7.31 (2.82–18.96) *P* < 0.01^*^	1.60 (0.73–3.55) *P* = 0.24	1.81 (0.49–6.60) *P* = 0.37	8.13 (3.34–19.81) *P* < 0.01^*^
TWC some days vs. never TWC	2.57 (1.13–5.86) *P* = 0.03^*^	5.78 (1.62–20.70) *P* < 0.01^*^	1.82 (0.75–4.42) *P* = 0.19	2.50 (1.26–4.98) *P* < 0.01^*^	1.25 (0.37–4.21) *P* = 71	4.34 (2.01–9.38) *P* < 0.01^*^

*NA* Not available since the sample size was too small for statistic calculation

^a^ Vegetable oil referred to palm oil and soybean oil

^b^ Size of restaurant is defined by number of kitchen strove: small restaurant is those with 1–3 strove; big restaurant is those with more than 3 strove

^*^ Statistically significantly difference with *P* <0.05

Some risk factors were associated with various health outcomes in a dose–response trend. A 1 h stay in the kitchen and the frequency of frying food were associated with most of the survey symptoms. Job period associated with “cough” in a dose–response style and working for more than 10 years increased the risk of “phlegm”, “wheeze”, “dyspnea”. An additional 10 h stay per week in the kitchen area increased the risk of “cough” by 15% and “wheeze” by 16%. Frying 351–745 dish per week predicted a risk of “cough” and “stuffy nose” and frying 746 or more predicted all the symptoms, except “dyspnea” and “severe dyspnea”. Fuel type cannot significantly predict any health outcome, statistically. Having a kitchen ventilation hood seemed to increase the risk of some symptoms but none were statistically significant. This point is discussed further elsewhere. Those workers who cook every day at home had a greater risk of “cough”, “wheeze”, “dyspnea” and “stuffy nose” than those who did not cook at home, and those who cooked at home only on some days had an increased risk of “stuffy nose”.

It was also found that there were many factors associated with symptoms in the past 30 days. Working in a restaurant with a separated kitchen room increased the risk of having a cold 3.46 times greater than those working in a restaurant without separated kitchen room (Table 4). Using vegetable oil increased risk of having a cold (OR = 2.8, *P* = 0.03). Size of restaurant predicted the risk of coughing (OR = 1.82, *P* = 0.04), having sputum (OR = 2.10, *P* = 0.02), sore throat (OR = 2.51, *P* < 0.01), allergic symptom (OR = 2.37, *P* = 0.05), and having any one symptoms (OR = 3.57, *P* < 0.01). This latter finding may also explain the greater risk inherent in having a separated kitchen room, which would then probably be a more confined space than if it was open to the restaurant area.

**Table 4:** Odds Ratios (OR) with p values for predictors for respiratory symptoms in the past 30 days of restaurant workers

Predictors	Coughing	Having sputum	Sore throat	Shortness of breath	Wheezing	Nasal irritation	Allergic symptom	Having a cold	Having any one symptoms
À la carte restaurant vs. other type	1.27 (0.75–2.18) *P* = 0.38	0.97 (0.53–1.78) *P* = 0.93	0.92 (0.51–1.67) *P* = 0.78	0.94 (0.35–2.50) *P* = 0.90	0.66 (0.28–1.58) *P* = .035	0.31 (0.08–1.22) *P* = 0.09	0.66 (0.29–1.53) *P* = 0.34	0.90 (0.49–1.65) *P* = 0.74	0.91 (0.51–1.61) *P* = 0.74
Noodle vs à la carte restaurant	1.68 (0.47–6.05) *P* = 0.43	1.61 (0.33–7.93) *P* = 0.56	0.93 (0.23–3.75) *P* = 0.92	NA	0.83 (0.09–7.28) *P* = 0.86	NA	0.42 (0.08–2.19) *P* = 0.30	0.94 (0.24–3.78) *P* = 0.94	(0.28–3.72) *P* = 0.98
Pre-prepared food vs à la carte restaurant	1.19 (0.29–4.88) *P* = 0.81	1.37 (0.24–7.81) *P* = 0.72	0.75 (0.16–3.55) *P* = 0.71	NA	1.96 (0.20–18.88) *P* = 0.56	NA	0.53 (0.08–3.42) *P* = 0.50	1.12 (0.25–5.13) *P* = 0.88	0.72 (0.17–2.98) *P* = 0.65
Papaya salad vs à la carte restaurant	1.50 (0.39–5.87) *P* = 0.56	2.05 (0.39–10.80) *P* = 0.40	1.22 (0.28–5.32) *P* = 0.79	NA	0.91 (0.09–8.94) *P* = 0.93	NA	0.63 (0.11–3.64) *P* = 0.61	(0.23–4.43) *P* = 0.99	1.70 (0.41–7.04) *P* = 0.47
Working 6–10 year vs 0–5 years	1.33 (0.72–2.45) *P* = 0.37	0.82 (0.40–1.70) *P* = 0.59	0.99 (0.49–1.98) *P* = 0.97	0.49 (0.14–1.71) *P* = 0.26	1.11 (0.41–2.98) *P* = 0.84	1.84 (0.39–8.82) *P* = 0.44	1.03 (0.39–2.69) *P* = 0.95	1.04 (0.51–2.13) *P* = 0.91	1.64 (0.84–3.20) *P* = 0.15
Working >10 year vs 0–5 years	0.80 (0.36–1.77) *P* = 0.58	2.03 (0.87–4.73) *P* = 0.10	1.46 (0.63–3.37) *P* = 0.38	0.86 (0.24–3.16) *P* = 0.82	0.65 (0.17–2.46) *P* = 0.52	2.30 (0.38–14.12) *P* = 0.37	0.83 (0.24–2.84) *P* = 0.77	1.86 (0.78–4.41) *P* = 0.16	1.12 (0.48–2.58) *P* = 0.80
Chef vs others	1.83 (1.03–3.27) *P* = 0.04^*^	1.77 (0.90–3.47) *P* = 0.10	0.95 (0.51–1.79) *P* = 0.88	0.63 (0.23–1.71) *P* = 0.36	1.25 (0.48–3.26) *P* = 0.65	2.14 (0.43–10.51) *P* = 0.35	1.09 (0.44–2.70) *P* = 0.85	1.09 (0.57–2.06) *P* = 0.80	1.59 (0.87–2.89) *P* = 0.13
Having vs not having kitchen area	1.35 (0.69–2.63) *P* = 0.38	1.51 (0.68–3.39) *P* = 0.32	1.99 (0.86–4.58) *P* = 0.11	0.80 (0.25–2.63) *P* = 0.72	2.73 (0.61–12.25) *P* = 0.19	2.28 (0.28–18.60) *P* = 0.44	NA	3.46 (1.29–9.27) *P* = 0.02^*^	1.73 (0.88–3.41) *P* = 0.12
Large vs. small restaurant^b^	1.82 (1.02–3.25) *P* = 0.04^*^	2.10 (1.12–3.94) *P* = 0.02^*^	2.51 (1.34–4.67) *P* < 0.01^*^	0.84 (0.28–2.51) *P* = 0.76	1.91 (0.78–4.65) *P* = 0.16	1.56 (0.42–5.74) *P* = 0.51	2.37 (1.01–5.54) *P* = 0.05^*^	1.61 (0.85–3.02) *P* = 0.14	3.57 (1.72–7.42) *P* < 0.01^*^
Vegetable oil vs. lard^a^	0.54 (0.28–1.04) *P* = 0.06	0.82 (0.40–1.69) *P* = 0.59	1.08 (0.52–2.25) *P* = 0.85	0.75 (0.25–2.31) *P* = 0.62	1.05 (0.36–3.06) *P* = 0.94	2.67 (0.32–22.09) *P* = 0.36	1.56 (0.50–4.89) *P* = 0.44	2.80 (1.11–6.97) *P* = 0.03^*^	1.30 (0.65–2.62) *P* = 0.45
Having vs. not having ventilation hood	0.67 (0.39–1.14) *P* = 0.14	0.75 (0.41–1.38) *P* = 0.36	1.15 (0.64–2.09) *P* = 0.64	0.75 (0.28–2.01) *P* = 0.57	1.08 (0.45–2.60) *P* = 0.86	0.41 (0.10–1.65) *P* = 0.21	0.92 (0.40–2.13) *P* = 0.85	0.83 (0.45–1.51) *P* = 0.53	0.73 (0.41–1.28) *P* = 0.27
Per 10 h increase in working in kitchen each week	1.07 (0.96–1.20) *P* = 0.19	1.14 (1.01–1.28) *P* = 0.04^*^	1.13 (1.00–1.27) *P* = 0.04^*^	1.26 (1.05–1.49) *P* = 0.01^*^	1.22 (1.04–1.42) *P* = 0.02^*^	1.16 (0.94–1.44) *P* = 0.17	1.03 (0.88–1.21) *P* = 0.70	1.05 (0.94–1.18) *P* = 0.38	1.16 (1.02–1.32) *P* = 0.02^*^
Fry 2–350 dishes per week vs. no fry	1.84 (0.83–4.04) *P* = 0.13	1.31 (0.54–3.17) *P* = 0.55	1.88 (0.74–4.78) *P* = 0.19	1.81 (0.43–7.68) *P* = 0.42	2.81 (0.54–14.63) *P* = 0.22	0.76 (0.10–5.79) *P* = 0.80	0.72 (0.19–2.75) *P* = 0.63	0.73 (0.30–1.77) *P* = 0.49	2.53 (1.05–6.05) *P* = 0.04^*^
Fry 351–745 dishes per week vs. no fry	1.80 (0.79–4.13) *P* = 0.16	0.94 (0.36–2.49) *P* = 0.90	2.44 (0.93–6.40) *P* = 0.07	1.47 (0.30–7.13) *P* = 0.63	5.19 (1.02–26.53) *P* = 0.05^*^	1.61 (0.25–10.25) *P* = 0.62	1.06 (0.28–4.02) *P* = 0.93	0.61 (0.23–1.59) *P* = 0.31	1.65 (0.69–3.95) *P* = 0.26
Fry ≥746 dishes per week v. no fry	1.43 (0.63–3.26) *P* = 0.40	1.78 (0.72–4.41) *P* = 0.21	1.52 (0.57–4.06) *P* = 0.40	0.53 (0.08–3.46) *P* = 0.51	1.76 (0.30–10.47) *P* = 0.53	1.28 (0.20–8.40) *P* = 0.80	0.82 (0.21–3.15) *P* = 0.77	1.29 (0.54–3.11) *P* = 0.57	1.37 (0.58–3.25) *P* = 0.47
Cooking every day at home vs. not cook at home	3.88 (1.35–11.14) *P* = 0.01^*^	4.60 (1.02–20.79) *P* = 0.05^*^	2.69 (0.75–9.72) *P* = 0.13	1.59 (0.19–13.48) *P* = 0.67	3.24 (0.40–26.16) *P* = 0.27	NA	3.56 (0.45–28.23) *P* = 0.23	4.19 (0.93–18.95) *P* = 0.06	5.62 (2.09–15.12) *P* < 0.01^*^
Cooking some days at home vs. not cook at home	2.93 (0.97–8.87) *P* = 0.06	3.74 (0.79–17.78) *P* = 0.10	2.98 (0.79–11.32) *P* = 0.11	2.94 (0.34–25.62) *P* = 0.33	1.67 (0.18–15.40) *P* = 0.65	NA	1.72 (0.19–15.70) *P* = 0.63	4.71 (1.00–22.20) *P* = 0.05^*^	5.54 (1.92–16.01) *P* < 0.01^*^
TWC every day vs. never TWC	3.66 (1.63–8.20) *P* < 0.01^*^	3.60 (1.35–9.61) *P* = 0.01^*^	3.00 (1.17–7.66) *P* = 0.02^*^	2.21 (0.41–11.84) *P* = 0.35	6.41 (1.34–30.64) *P* = 0.02^*^	1.18 (0.18–7.69) *P* = 0.86	4.39 (1.14–16.85) *P* = 0.03^*^	1.47 (0.59–3.65) *P* = 0.41	6.35 (2.56–15.78) *P* < 0.01^*^
TWC some days vs. never TWC	2.33 (1.16–4.67) *P* = 0.02^*^	2.82 (1.15–6.95) *P* = 0.02^*^	2.47 (1.04–5.83) *P* = 0.04^*^	2.50 (0.52–12.00) *P* = 0.25	2.01 (0.41–9.84) *P* = 0.39	1.22 (0.23–6.51) *P* = 0.82	1.52 (0.39–5.85) *P* = 0.55	1.77 (0.81–3.87) *P* = 0.15	2.79 (1.42–5.49) <0.01^*^

*NA* Not available since the sample size was too small for statistical calculation

^a^ Vegetable oil refers to palm oil and bean oil

^b^ Size of restaurant is defined by number of kitchen stoves. Small restaurants are those with 1–3 stoves, and big restaurants are those with more than 3 stoves

^*^ Statistically significantly different with *P* <0.05

Every additional 10 h stay in the kitchen per week increased the risk of having sputum in the past 30 days by 14%, sore throat by 13%, shortness of breath by 26%, wheezing by 22% and having any one of these symptoms by 16%. Those who fry 2–350 dishes per week had a higher risk of “having any one symptom” than those who did not fry any dishes (OR = 2.53) and those frying 351–745 dishes per week had an even higher risk of wheezing (OR = 5.19). Cooking every day at home increased the risk of coughing (OR = 3.88, *P* < 0.01), having sputum (OR = 4.60, *P* = 0.05) and having at least one symptom (OR = 5.62, *P* < 0.01), while cooking some days at home can predict only the risk of having a cold (OR = 4.71, *P* = 0.05) and having any the symptoms (OR = 5.54, *P* < 0.01). TWC every day can predict all of the symptoms, except shortness of breath, nasal irritation and having a cold, while TWC some days only predicted the risk of coughing, having sputum and sore throat. Again, type of restaurant and having a ventilation hood failed to predict any survey symptoms.

## Discussion

Compared with the control group, restaurant workers had a higher risk of dyspnea, stuffy nose, cough, wheeze, phlegm and many of the survey symptoms in the past 30 days (Table 2). This finding is in line with a previous study in Norway [17] and Nigeria [18]. Based on data from 239 workers in 67 kitchens in Norway, Svendsen et al. [17] found exposure to kitchen smoke to be associated with an increased risk of dyspnea (RR = 4.1; 95%CI 2.7, 6.3), severe dyspnea (RR = 2.9; 95%CI 1.5, 5.7) and other health symptoms related to work (RR = 4.3; 95%CI 2.7, 6.7) among women employees. Among male employees, only dyspnea (RR = 1.8; 95%CI 1.4, 2.3) and other health symptoms (RR = 4.3; 95%CI 2.7, 6.7) was found to be significantly increase. A recent study in Nigeria [18] also reported a significant increase in chest tightness (OR = 3.1, *P* = 0.0.04), wheeze (OR = 1.2, *P* = 0.04) and nasal congestion (OR = 1.2, *P* = 0.02) among certain types of restaurant worker in Nigeria. It is well established that kitchen smoke contains thousands of toxic chemicals and many of them are respiratory irritants, including nitrogen dioxide, particulate matter, acrolein and formaldehyde [1, 2]. Exposure to these pollutants is known to damage the respiratory tract and cause the problematic respiratory symptoms [4, 5, 11].

We compared the respiratory symptoms of restaurant workers from different cultures and working practices, described in the literature, with the results from our. Norway had a higher RR for dyspnea (4.1 vs 1.9 for our study) and severe dyspnea (2.9 vs our RR of 1.4) among women group, but lower RR of both symptom among men group (Our study 3.1 vs Norwegian study 1.8 for dyspnea; 1.4 vs 0.6 for severe dyspnea). The divergence of RR mainly comes from the difference of the symptom prevalence among the control groups of the two studies. While the prevalence of dyspnea among the sample of restaurant workers (52.3% in the Norwegian study vs 43.5% for our study) was comparable, the prevalence of dyspnea in the control group in our study was much higher than that in Norway (27.5% vs 10.6%). It is probable that Thai women are likely to have a poorer health background and greater exposure to other source of pollution, such as ambient air pollution and kitchen smoke, than women in Norway. Our result showed that 85.0% of women in our control group reported cooking at home (Table 1). The lower RR between the male groups in the two surveys can be explained by an unusually high rate of dyspnea (25.5%) and severe dyspnea (16.8%) in Norway was explained as either employment in polluted work areas, and/or selection bias [17]. In the comparison between our study and the Nigerian study [18], the RR of “wheeze” was 2.1 among women and 9.9 among men, and for “stuffy nose” was 1.7 among women and 3.1 among men, with the Nigerian study showing the higher rates. The OR was used in the Nigerian study for a particular type of restaurant worker (Mai suyas worker) which resulted in the RR figures being higher than the OR, which were 1.2 for both “wheeze” and “stuffy nose” symptoms. Differences in background health, pollution level and working practices might account for the disparity.

This study also found many of the symptom in the past 30 days to be associated with restaurant work. Women restaurant workers reported a higher rate of shortness of breath (RR = 2.1; 95%CI 1.0–4.4) and having any one symptom (RR = 1.3, 95%CI 1.1, 1.6) (Table 2). Among men, a significantly increased risk was found for coughing, having a cold, sore throat, having sputum, wheezing and having any one symptom. This result was also seen in a recent study in Hong Kong [19], which reported a higher prevalence of these symptoms, and OR, of workers in a gas fueled kitchen compared to those in electric kitchens. For coughing prevalence ratio between restaurant workers and the control group was 11.2 vs 9.6 (OR = 1.1; 95%CI 0.5, 2.4), phlegm 16.5% vs 122% (OR = 1.3; 95%CI 0.6, 2.6), runny nose 11.2% vs 11.3% (OR = 1.1; 95%CI 0.5, 2.3) and sore throat 13.7% vs 6.1% (OR = 2.3; 95%CI 1.0, 5.7). However, a higher rate and RR for coughing, phlegm and sore throat, was found in our study (see Table 2). The difference in cooking style, work practices and work environment might be the main source for the variance. Restaurants in Hong Kong are likely to have higher working standards with more modern equipment using cleaner fuels than those in Thailand which are mainly small restaurants in rural areas. In addition, this study employed the general public as the control group whereas the previous studies using workers in kitchen with electric cooking equipment as the control.

When comparing women to men restaurant workers, there was a difference in the prevalence and RR of respiratory symptoms. Women had a higher prevalence of “dyspnea” (52.3% of women vs 30.7% of men) and “severe dyspnea” (11.4% women vs 6.7% men) but men had more “wheeze” (38.7% vs 25.5% for women) and “phlegm” (25.3% vs 14.4% for women) (Table 2). The elevated levels among women of dyspnea and severe dyspnea, which are signs of chronic pulmonary disease, was in line with previous findings of greater susceptibility to COPD among women [23]: cooking at home might be the source of this bias. The Norwegian study [17] also showed a similar outcome. A higher rate of “wheeze” and “phlegm” among men can be explained by higher number of men smoking cigarettes (53.5% of men vs 3.2% of women). A higher RR among men for “dyspnea” (1.9 for women vs 3.1 for men), “stuffy nose” (1.7 for women vs 3.1 for men) and “wheeze” (2.1 for women vs 9.9 for men) came mainly from the low rate of the symptoms among the control group. For “cough”, the prevalence was similar for both women and men workers but the RR was higher in the women workers due to a lower rate among the members of the control group. Higher cigarette consumption by the men might explain this finding.

Several factors can predict chronic symptoms and symptoms in the past 30 days. Duration of working can predict the risk of most of chronic survey symptoms, a similar finding to [18]. Every 10 additional hours per week working in a kitchen area increases the risk of “cough” by 15%, “wheeze” by 16%, having sputum in the past 30 days by 14%, sore throat in the past 30 days by 13%, shortness of breath in the past 30 days by 26%, wheezing in the past 30 days by 22% and having any one symptoms in the past 30 days by 16%. It was also reported in [17] that there was a 20% increase in dyspnea and other symptoms in connection with work among kitchen workers in Norway. The number of fried dishes prepared per week can also predict most of the survey symptoms and in a dose–response trend for “cough”, “phlegm” and “wheeze” (Table 3). It was also found that, compared with lard, using vegetable oil can predict risk of “stuffy nose” and having a cold in the past 30 days. This results in line with recent study that suggested that frying food with vegetable oil will release more aldehyde and is therefore considered more harmful than frying with lard [24]. Cooking at home can predict many symptoms, including “cough”, “wheeze”, “dyspnea”, “stuffy nose”, coughing in the past 30 days, having sputum in the past 30 days and having a cold in the past 30 days. This could bias the result toward the null. This study also confirmed the previous finding that “tears while cooking” is a good indicator for smoke exposure [25]. TWC can predict most of the survey symptoms.

It was an unexpected result that working in an à la carte restaurant which has more frying activity was not associated with a rise in the prevalence of any of the health symptoms as compared with working in the other types of restaurant. This implies that working in the other types of restaurants, which serve pre-prepared, noodle and papaya salad, might also risk respiratory hazards but from types of activities other than frying. As well, workers involved with pre-prepared food might also be involved in cooking during the food preparation process. As the data showed, the 49 workers serving pre-prepared food came from 31 restaurants and this implied that the shop owner both cooked and served the food by themselves. Workers in a noodle restaurant, although not involve in frying activity, are often exposed to fuel smoke and vapors from boiling soup which are also harmful [7]. Papaya salad restaurant also often sell grilled or fried meat, chicken, fish, or pork and might be exposed to toxic smoke from broiling activity. Our study showed that health outcomes were influenced not only by frying activity but also other variables, such as cooking method, fuel and oil use, type of kitchen and ventilation systems. Future research should employ quantitative robot indicators such as fried dishes per week, hours working in the kitchen, or TWC as an exposure index.

Another unusual finding was that having a ventillation hood did not lower risk of any symptoms and that having separated kitchen room actually increased the risk of “wheeze”, “stuffy nose” and having a cold in the past 30 days. The possible explanation of these two phenomena is that having a separate kitchen room might limit natural air ventilation and caused pollution to be accumulated. It was also observed that installed ventilation hoods were often not in use or were not well designed and didn’t function in an effective way. Data on ventilation hoods alone may not indicate better ventilation, and more information on the use and efficiency of the ventilation system is also needed.

There were some limitations in this study. Information on health symptoms was self-reported which could introduce information bias. However, by using a standard questionnaire and well-trained qualified staff, the error should be minimized with no relevant difference in distribution between the study group and the control group. If anything, this possible bias would lower, not increase, the estimated risk. When asking about symptoms in the past, recall bias might also occur because concern and ability to remember the health problems of study and control group may not be the same for the participants in each group. However, by asking about health symptoms in the immediate past 30 days, the problem was expected to be minimal. Another bias, the so called “healthy worker effect”, is expected in this type of occupational health study because, normally, the study participants tend to be stronger than the general population [26]. This can be explained by the fact that those workers who become ill will be forced, or will volunteer, to leave, leaving only healthy individuals to be selected for study. This type of bias will lead to underestimation of the impact.

## Conclusions

This study is among a very few to investigate health problems of restaurant workers arising from their work. Compared with the general population, restaurant workers have a much higher prevalence of both chronic and acute respiratory symptoms. A number of factors were identified as predictive factors for a variety of health problems, particularly respiratory problems. The list of the factors includes job description, job period, size of restaurant, kitchen location, cooking oil, hours of stay in the kitchen area, frequency of fried dishes, frequency of occurrence of TWC, and additional cooking at home. It is suggested that more risk factors may well be identified if further studies using a larger sample size, under a greater variety of working environments, were conducted. Generally, workplace health and safety measures providing greater health protection of restaurant workers are urgently needed and the issue should receive more public attention.

## Abbreviations

Cr (VI): Hexavalent chromium
Dish-year: Number of dishes cooked in a year
OR: Odd ratio
PM10: Particulate with aerodynamic diameter smaller than 10 μm
PM2.5: Particulate with aerodynamic diameter smaller than 2.5 μm
RR: Rate Ratio
TWC: Tears while cooking
